# Increased transmembrane protein 119 (TMEM119) levels in the cerebrospinal fluid of patients with mild cognitive impairment due to Alzheimer's disease suggest early microglial involvement

**DOI:** 10.1002/dad2.70240

**Published:** 2025-12-31

**Authors:** Paula Klassen, Christoforos Alexudis, Veronika Klose, Nerea Gómez de San José, André Huss, Franziska Bachhuber, Önder Soylu, Badrieh Fazeli, Deborah Erhart, Mona Laible, Sarah Anderl‐Straub, Sarah Jesse, Markus Otto, Albert C. Ludolph, Hayrettin Tumani, Steffen Halbgebauer

**Affiliations:** ^1^ German Center for Neurodegenerative Diseases (DZNE e.V.) Ulm Germany; ^2^ Department of Neurology Ulm University Hospital Ulm Germany; ^3^ Department of Neurology University Hospital Halle Halle (Saale) Germany

**Keywords:** Alzheimer's disease, biomarkers, cerebrospinal fluid, microglia, neuroinflammation

## Abstract

**Introduction:**

We aimed to evaluate the potential of the microglial marker transmembrane protein 119 (TMEM119) in the cerebrospinal fluid (CSF) as a (differential) diagnostic biomarker for neurodegenerative diseases.

**Methods:**

Following assay validation, we used enzyme‐linked immunosorbent assay to measure CSF TMEM119 in 174 patients from six diagnostic groups: Alzheimer's disease (AD, *n *= 35), amyotrophic lateral sclerosis (ALS, *n *= 33), cerebral microangiopathy (CM, *n *= 25), frontotemporal lobar degeneration (FTLD, *n *= 28), Lewy body diseases (*n *= 21), and non‐neurodegenerative controls (*n *= 33).

**Results:**

CSF TMEM119 levels were elevated in the AD group compared to the control (*p *= 0.004), CM (*p *= 0.005), and FTLD (*p *= 0.023) groups. Levels were higher in both mild cognitive impairment (MCI‐AD) and dementia (ADD) subgroups when compared to controls. For the discrimination of AD from controls, the area under the curve (AUC) was 0.78.

**Discussion:**

Our results indicate that CSF TMEM119 may have potential as a biomarker representing microglial involvement in early and later stages of AD.

**Highlights:**

- Elevated levels of TMEM119 were observed in the CSF of patients with AD.
- Increased CSF TMEM119 was seen in MCI‐AD patients compared to controls.
- Elevated levels in MCI‐AD underscore early microglial involvement in AD.
- In the AD group, an association was found between CSF TMEM119 and CSF total tau.
- CSF TMEM119 may provide valuable information on neuroinflammation.

## 1 BACKGROUND

Alzheimer's disease (AD) is recognized as the most common cause of dementia.^1^ It is characterized by memory impairment and progressive cognitive decline and has been shown to have a preclinical phase that can last several decades.^2^, ^3^ Major pathological hallmarks of AD include the accumulation of amyloid beta (Aβ) plaques and neurofibrillary tangles.^4^ The measurement of levels of Aβ peptide 1–42 (Aβ1–42) and tau protein in cerebrospinal fluid (CSF) has been incorporated into the current diagnostic criteria for AD.^5^ Although these markers of amyloid and tau are indicative of the major features of the disease, they do not represent the full spectrum of pathological processes occurring in the brains of individuals with AD, such as neuroinflammation mediated by astro‐ and microglial activation. Neuroinflammation has emerged as a key player in AD pathogenesis.^6^ Microglia, the resident immune cells of the brain, contribute to the maintenance of homeostasis in the brain and mediate inflammatory processes.^7^ In early AD, it has been suggested that microglia become activated and assist in the clearance of Aβ from the brain, thereby playing a protective role.^8^ However, it is hypothesized that in AD, dystrophic microglia may also contribute to synaptic loss and neurodegeneration.^9^ A number of genetic mutations related to microglia have been identified as potential risk factors for AD, indicating that microglial function can play a role in the development of the disease.^10^, ^11^ Mutations in triggering receptor expressed in myeloid cells 2 (TREM2), expressed by microglia and other macrophages, have been linked to an increased risk of AD,^12^ and several studies on the soluble form of the protein TREM2 (sTREM2) have revealed increased CSF concentrations in patients with AD compared to controls.^13^, ^14^, ^15^, ^16^ The development of fluid‐based biomarkers related to microglia could therefore provide valuable information about another important facet of AD pathology.

Transmembrane protein 119 (TMEM119) is a cell‐surface protein that has been identified as a predominantly microglia‐specific marker in the central nervous system (CNS).^17^, ^18^ Its specificity can help to distinguish microglia from peripherally derived macrophages that may also express TREM2.^19^, ^20^ TMEM119 is associated with homeostatic microglia, as a downregulation of its expression has been shown in disease‐associated microglia (DAM) states.^21^, ^22^ In the brain parenchyma, an increase in *TMEM119* mRNA expression levels has been observed in AD brains when compared to non‐AD brains, though this has not been reflected at the protein level.^18^, ^23^, ^24^, ^25^ A significant increase in the density of TMEM119‐positive cells has been observed in the motor cortex and subcortical white matter of patients with amyotrophic lateral sclerosis (ALS) compared to controls.^26^ In the CSF, TMEM119‐positive cells have been detected using immunocytochemistry in patients with aneurysmal subarachnoid hemorrhage and individuals who died as a result of traumatic brain injury.^27^, ^28^ It remains an open question whether the differences in mRNA and protein expression at the tissue level may be reflected in the CSF.

In this study, a commercially available enzyme‐linked immunosorbent assay (ELISA) kit was used to quantify levels of TMEM119 in the CSF of patients with AD, Lewy body diseases (LBD), ALS, frontotemporal lobar degeneration (FTLD), cerebral microangiopathy (CM), and in individuals with no known neurodegenerative diseases, to determine whether group differences in CSF TMEM119 may be observable. To our knowledge, this is the first study in which an immunoassay was used to compare CSF levels of TMEM119 in patients with AD and other neurological diseases.

## 2 MATERIALS AND METHODS

### 2.1 Patient selection

In this study, CSF samples from 174 patients from six diagnostic groups were examined. The samples were collected between 2011 and 2021 in the Department of Neurology in Ulm. The study was approved by the Ethics Committee of Ulm University (approval number 20/10) and conducted following the Declaration of Helsinki. All patients or their legal proxies gave their written informed consent for inclusion in the study. For the quantification of CSF levels of TMEM119, samples from patients with AD (*n *= 35), ALS (*n *= 33), CM (*n *= 25), FTLD (*n *= 28), LBD (*n *= 21), and non‐neurodegenerative controls (*n *= 33) were selected.

AD patients were diagnosed according to the International Working Group 2 criteria.^29^ AD inclusion criteria included CSF Aβ 1–42 levels < 550 pg/mL and CSF phosphorylated tau 181 (p‐tau181) levels ≥ 62 pg/mL. The cutpoints were derived using a standardized in‐house optimization procedure based on a well‐characterized cohort consisting of (i) clinically and biomarker‐validated AD cases and (ii) age‐matched symptomatic controls without neurodegenerative or inflammatory CNS pathology (e.g., tension‐type headache, benign paroxysmal positional vertigo, and non‐specific sensory symptoms), all with normal brain MRI and unremarkable routine CSF findings. Receiver operating characteristic (ROC) analyses were performed using AD versus non‐neurodegenerative controls as the classification contrast, and Youden's Index (sensitivity + specificity – 1) was used to determine optimal thresholds. Patients in the ALS group had been diagnosed with definite or probable ALS according to the revised El Escorial criteria.^30^ The CM group consisted of patients who had no known neurodegenerative disease, but who were given a Fazekas score of 2 to 3 following magnetic resonance imaging (MRI).^31^ The 28 patients with FTLD consisted of 15 patients with confirmed or probable behavioral variant FTLD (bvFTLD) and 13 with primary progressive aphasia (PPA). The patients in the FTLD group were diagnosed according to accepted international criteria.^32^, ^33^ The LBD spectrum group comprised patients who had been diagnosed by specialists according to the UK PD Society Brain Bank criteria.^34^ The individuals included in the control group displayed no clinical or radiological signs of neurodegeneration. All patients underwent a lumbar puncture in order to exclude acute inflammation of the central nervous system.

**RESEARCH IN CONTEXT**

1. **Systematic review**: Previous studies identified TMEM119 as a predominantly microglia‐specific marker in the CNS, with changes in mRNA expression seen in the brains of individuals with AD. However, there are limited reports assessing its utility as a biomarker in cerebrospinal fluid.
2. **Interpretation**: Our findings position TMEM119 as a promising candidate biomarker that may complement the established ATN framework by capturing neuroinflammatory dynamics. Beyond its diagnostic utility, TMEM119 holds potential as a translational tool for patient stratification and for monitoring therapeutic responses targeting microglial pathways in AD.
3. **Future directions**: Further research may include longitudinal studies and comparisons with other microglial markers to help discern the potential benefit of using cerebrospinal fluid (CSF) TMEM119 as a microglial biomarker in AD.

For part of the analysis, the AD group was stratified into AD‐dementia (ADD, *n *= 23) and mild cognitive impairment (MCI‐AD, *n *= 12) subgroups. The stratification into either subgroup was based on the clinical dementia rating sum of boxes score (CDR‐SOB).^35^ Patients with a CDR‐SOB below 2.5 were classified as having MCI‐AD, while those with scores of 2.5 or higher were classified as having ADD.^36^

### 2.2 CSF collection and analysis

The CSF samples used in this study were collected via lumbar puncture and centrifuged at 2000 × g for 10 min. Supernatants were aliquoted and stored at –80°C within 30 min. CSF levels of total tau (t‐tau), p‐tau181, and Aβ1–42 for the patients in the AD group were quantified using commercially available ELISA kits (Fujirebio, Hanover, Germany). The albumin quotient (QAlb), representing the ratio of CSF albumin to serum albumin, was determined as part of routine diagnostics.

The quantification of CSF TMEM119 was done using a commercially available ELISA kit (abx549911, Abbexa, Cambridge, UK). The assay was performed according to the manufacturer's instructions apart from the wash steps, where a volume of 300 µL was used. CSF samples were diluted using a dilution factor of 1:4. Two CSF quality control samples were included on each plate for the cohort measurement.

### 2.3 Partial validation of commercial ELISA kit

In addition to the cohort measurement, a partial validation of the ELISA kit for use with CSF samples was completed based on previously described guidelines.^37^ Inter‐ and intra‐assay reproducibility were assessed by the analysis of two CSF samples run in sextuplicate on one plate and duplicate on two others. The limit of detection (LOD) and lower limit of quantification (LLOQ) were determined by running 16 blank samples. The LOD and LLOQ were calculated as three standard deviations (SD) and 10 SD above the mean of the blanks, respectively. Two samples were measured undiluted and diluted 1:2, 1:4, 1:6, 1:8, 1:16, and 1:32 in order to assess parallelism. Three concentrations (300, 800, and 1500 pg/mL) of the standard protein from the ELISA kit were spiked into CSF samples in order to evaluate the recovery of the spiked protein. Recovery was calculated according to the formula described by Andreasson et al.^37^

To test for cross‐reactivity to highly abundant proteins, CSF samples were spiked with physiological CSF concentrations of human immunoglobulin G (IgG, 30 µg/mL) and human albumin (200 µg/mL). TMEM119 concentrations were compared between the spiked and unspiked samples. Stability testing at 4°C and room temperature and for up to five additional freeze‐and‐thaw cycles was additionally performed.

### 2.4 Statistical analysis

All statistical analyses were performed using R Statistical Software (version 4.3.1; R Core Team, 2023) on RStudio (version 2023.9.0.463). The Shapiro‐Wilk tests were used to determine the distribution of data among the different diagnostic groups. For comparisons between multiple groups, a Kruskal–Wallis test followed by Dunn's post hoc test with Bonferroni correction was applied. Following log_2_ transformation of CSF TMEM119 levels, an analysis of covariance (ANCOVA) and post hoc Tukey Honestly Significant Difference (HSD) test were performed to control for the influence of sex. A Wilcoxon rank‐sum test was used for comparisons between two groups. A χ^2^ test was used to determine differences in sex distribution between groups. Spearman's rank correlation coefficient was calculated, and simple linear regression was conducted to evaluate associations between various parameters. ROC analysis was performed to visualize the potential of TMEM119 for discrimination between patients with AD and the other diagnostic groups. Youden's Index was maximized in order to determine optimal cut‐off levels. A *p* value < 0.05 was considered statistically significant for all analyses.

## 3 RESULTS

### 3.1 Performance of TMEM119 ELISA kit

The intra‐ and inter‐assay coefficients of variation for CSF TMEM119 were determined to be 3.6% and 3.8%, respectively. A LOD of 0.110 ng/mL and a LLOQ of 0.135 ng/mL were calculated based on the mean and standard deviation of 16 blank samples. The evaluation of the assay for parallelism revealed that CSF samples were stable between dilutions of 1:2 and 1:8 (Figure S1). The recovery rates for the three spiking concentrations fell between 89.6% and 94.9% (Table S1). No evidence of a cross‐reaction was detected with either human IgG or human albumin. Finally, stability testing of CSF samples showed that TMEM119 levels remained within a deviation of 20% when compared to a reference sample after up to 5 days at 4°C or room temperature and for up to five additional freeze‐and‐thaw cycles (Figure S2).

### 3.2 Demographic and clinical features

In this study, the CSF of a total of 174 patients was evaluated. Patient samples were divided based on diagnosis, with 35 AD, 33 ALS, 24 CM, 28 FTLD, 21 LBD, and 33 control patients making up the different groups. The clinical and demographic parameters of the diagnostic groups are summarized in Table 1. No significant difference in age (*p = *0.368) or QAlb (*p = *0.136) was observed among the different diagnostic groups. The overall sex distribution was found to differ significantly between diagnostic groups (*p = *0.026). In the control group, there was no significant correlation of CSF TMEM119 with age (*R* = 0.30, *p = *0.092) or QAlb (*R* = 0.23, *p = *0.206), and no significant difference was observed between males and females (*p = *0.769). Associations between CSF TMEM119 and age, QAlb, and sex are shown for each of the diagnostic groups, as well as for the overall cohort in Figures S3–S5, respectively. No significant association with age or QAlb was found in any of the diagnostic groups. CSF TMEM119 was significantly higher in males than females in the ALS group (*p *= 0.018) but did not differ significantly between sexes in the other diagnostic groups.

**TABLE 1:** Demographic and clinical characteristics of the diagnostic groups.

	Control (*n *= 33^*^)	AD (*n *= 35^*^)	ALS (*n *= 33^*^)	CM (*n *= 24^*^)	FTLD (*n *= 28^*^)	LBD (*n *= 21^**^)	*p*‐value^**^
Age [years]	67 (62, 75)	73 (65, 78)	68 (64, 73)	71 (64, 79)	68 (64, 72)	71 (67, 73)	0.368
Sex							0.026
Female	23 (70%)	21 (60%)	13 (39%)	15 (63%)	15 (54%)	6 (29%)	
Male	10 (30%)	14 (40%)	20 (61%)	9 (38%)	13 (46%)	15 (71%)	
CSF TMEM119 [ng/mL]	2.04 (1.64, 2.54)	2.74 (2.44, 3.38)	2.53 (1.79, 3.09)	1.86 (1.65, 2.40)	2.16 (1.87, 2.49)	2.21 (1.83, 2.69)	0.001
QAlb × 10^−3^	5.75 (4.53, 6.57)	5.52 (4.41, 7.29)	6.52 (5.48, 8.14)	6.48 (4.72, 7.65)	6.66 (5.26, 8.21)	6.66 (5.34, 8.41)	0.136
CSF Aβ1–42 [pg/mL]	1009 (828, 1167)	435 (359, 500)	1103 (884, 1234)	1078 (805, 1239)	969 (678, 1155)	900 (613, 1100)	<0.001
CSF t‐tau [pg/mL]	253 (209, 327)	727 (634, 991)	278 (192, 380)	266 (186, 313)	381 (277, 595)	297 (251, 376)	<0.001
CSF p‐tau [pg/mL]	58 (44, 81)	118 (97, 144)	41 (27, 60)	45 (32, 70)	48 (39, 59)	53 (46, 62)	<0.001

*Note*: CSF Aβ1–42 and CSF t‐tau data were missing for 12 controls, two ALS, eight CM, and three FTD patients. CSF p‐tau data were missing for 13 controls, two ALS, eight CM, four FTD, and five LBD patients.

Abbreviations: AD, Alzheimer's disease; ALS, amyotrophic lateral sclerosis; CM, cerebral microangiopathy; CSF, cerebrospinal fluid; FTLD, frontotemporal lobar degeneration; IQR, interquartile range; LBD, Lewy body diseases; QAlb, albumin CSF/serum quotient; TMEM119, transmembrane protein 119.

* Median (IQR); *n* (%).

** Kruskal–Wallis rank‐sum test, Pearson's chi‐squared test.

### 3.3 CSF TMEM119 concentrations in the diagnostic groups

**FIGURE 1:**
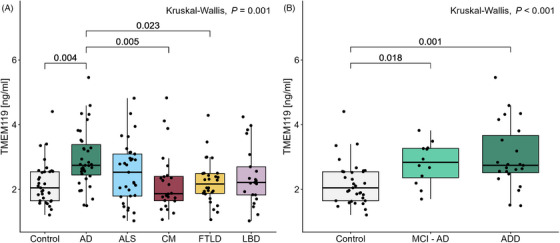
Levels of CSF TMEM119 across diagnostic groups. (A) CSF TMEM119 levels in all diagnostic groups. Levels in the AD group were significantly different than in the control (*p* = 0.004), CM (*p* = 0.005), and FTLD (*p* = 0.023) groups. (B) CSF TMEM119 levels in control and stratified AD groups. TMEM119 levels were elevated in the MCI‐AD group (*n* = 12) compared to controls (*p* = 0.018) and in the ADD group (*n* = 23) compared to controls (*p* = 0.001). Concentrations are displayed as box plots, with points representing individual patients. The median and IQR are illustrated, with whiskers extending to ±1.5 × IQR. AD, Alzheimer's disease; ADD, Alzheimer's disease dementia; ALS, amyotrophic lateral sclerosis; CSF, cerebrospinal fluid; CM, cerebral microangiopathy; FTLD, frontotemporal lobar degeneration; IQR, interquartile range; LBD, Lewy body diseases; MCI‐AD, mild cognitive impairment‐Alzheimer's disease; TMEM119, transmembrane protein 119.

CSF TMEM119 levels were found to be significantly higher in the AD group when compared to the control (*p = *0.004), CM (*p = *0.005), and FTLD (*p = *0.023) groups (Figure 1). The second‐highest CSF TMEM119 levels were observed in the ALS group, with a median concentration of 2.53 ng/mL (IQR: 1.79 to 3.09). When the AD group was stratified into MCI‐AD and ADD groups, the CSF TMEM119 concentration was found to be significantly higher in both the MCI‐AD (*p = *0.018) and ADD (*p *= 0.001) groups compared to the control group. An ANCOVA was conducted on log_2_‐transformed CSF TMEM119 levels to control for sex, *F*(5, 167) = 3.89, *p *= 0.002 (Table S2). A post hoc Tukey HSD test revealed significantly higher levels of CSF TMEM119 in the AD group compared to the control (*p = *0.008), CM (*p = *0.008), and FTLD (*p = *0.013) groups (Table S3). For the comparison between the control and AD subgroups, sex was not shown to have a significant effect on the outcome, *F*(1, 64) = 0.10, *p *= 0.748 (Table S4).

### 3.4 Association of TMEM119 with dementia scores and AD biomarkers (AD groups only)

**FIGURE 2:**
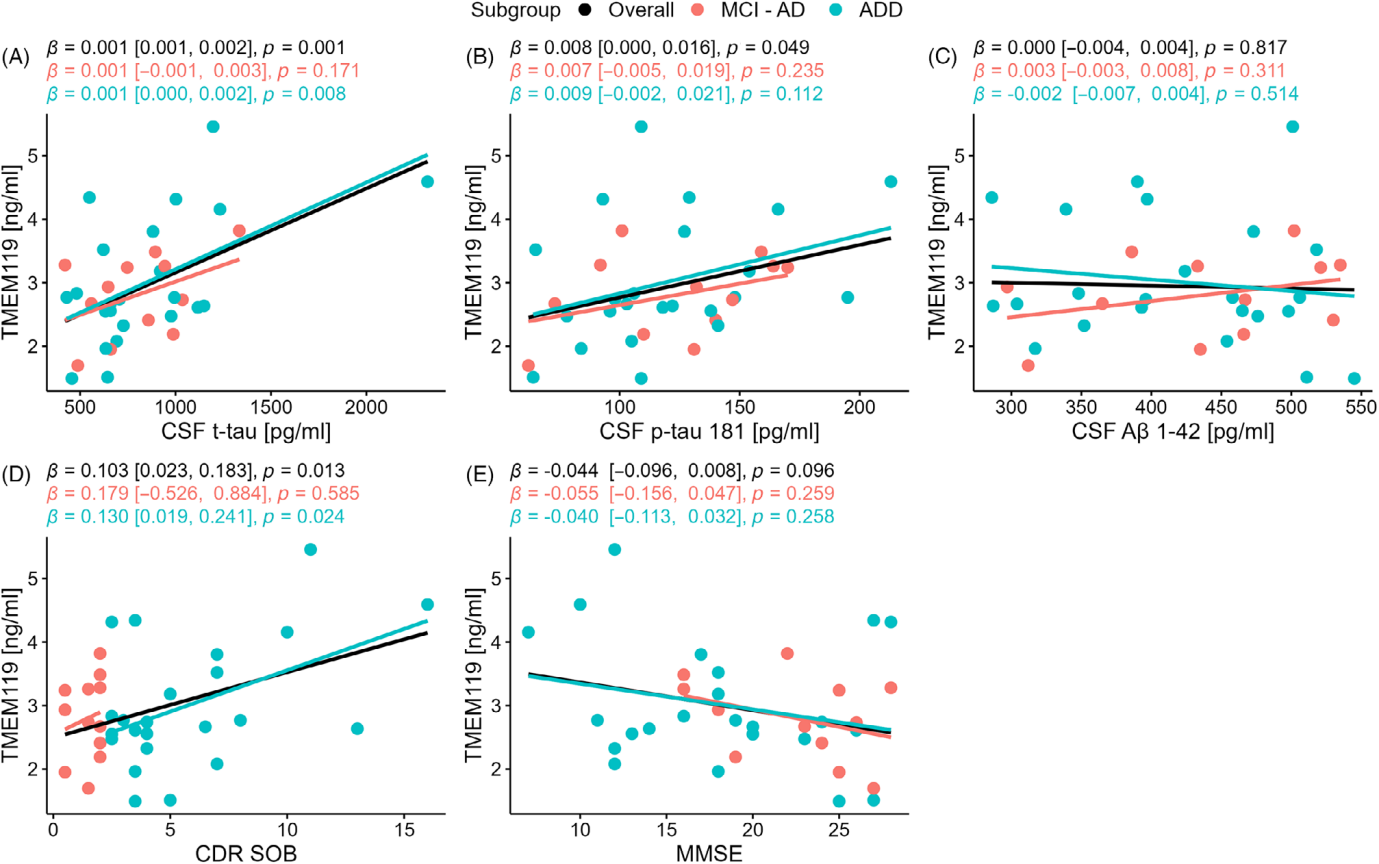
Simple linear regression analysis between CSF, TMEM119, and AD parameters in 35 AD patients. Regression lines and statistics are in black for the overall cohort (*n* = 35), pink for the MCI‐AD subgroup (*n* = 12), and blue for the ADD subgroup (*n* = 23). Numbers in brackets represent the 95% CI. (A) Association between CSF, TMEM119, and CSF t‐tau. (B) Association between CSF, TMEM119, and CSF p‐tau181. (C) Association between CSF, TMEM119, and CSF Aβ1‐42. (D) Association between CSF, TMEM119, and CDR‐SOB score. (E) Association between CSF, TMEM119, and MMSE score. Aβ, amyloid‐β; AD, Alzheimer's disease; ADD, Alzheimer's disease dementia; CDR‐SOB, clinical dementia rating sum of boxes; CSF, cerebrospinal fluid; CI, confidence interval; MCI‐AD, mild cognitive impairment‐Alzheimer's disease; MMSE, Mini‐Mental State Examination; p‐tau181, phosphorylated tau 181; t‐tau, total tau; TMEM119, transmembrane protein 119.

The AD group was subdivided into MCI‐AD and ADD groups. Clinical characteristics of the overall group specific to AD are summarized in Table 2. Linear regression analysis was performed to identify associations between CSF TMEM119 and specific AD parameters in the overall group and in the MCI‐AD and ADD subgroups (Figure 2). In the overall AD group, CDR‐SOB (*β *= 0.103, *p *= 0.013, *R* ^2^ = 0.174), CSF t‐tau (*β *= 0.001, *p *= 0.001, *R* ^2^ = 0.275), and CSF p‐tau (*β *= 0.008, *p *= 0.049, *R* ^2^ = 0.113) were significantly associated with CSF TMEM119 levels. In the ADD subgroup, there was a significant association between CSF TMEM119 and CDR‐SOB (*β *= 0.130, *p *= 0.024, *R* ^2^ = 0.221) and CSF t‐tau (*β *= 0.001, *p *= 0.008, *R* ^2^ = 0.293). No significant associations between CSF TMEM119 and specific AD parameters were observed in the MCI‐AD subgroup (Figure 2).

**TABLE 2:** Clinical characteristics of the Alzheimer's disease group.

	Overall (*n *= 35^*^)	MCI‐AD (*n *= 12^*^)	ADD (*n *= 23^*^)	*P* value^**^ (MCI‐AD vs ADD)
Age [years]	73 (65, 78)	71 (66, 78)	74 (66, 78)	0.745
Sex				0.884
Female	21 (60%)	7 (58%)	14 (61%)	
Male	14 (40%)	5 (42%)	9 (39%)	
CDR‐SOB	3.5 (2.0, 5.8)	1.8 (1.3, 2.0)	4.0 (3.5, 7.0)	<0.001
MMSE	19 (16, 25)	24 (19, 25)	18 (13, 24)	0.062
CSF t‐tau [pg/mL]	727 (634, 991)	803 (625, 955)	706 (634, 998)	0.960
CSF p‐tau181 [pg/mL]	118 (97, 144)	132 (99, 150)	109 (97, 140)	0.614
CSF Aβ1–42 [pg/mL]	435 (359, 500)	451 (381, 507)	424 (350, 487)	0.504

*Note*: Overall refers to all AD patients.

Abbreviations: AD, Alzheimer's disease; ADD, Alzheimer's disease dementia; Aβ, amyloid beta; CDR‐SOB, clinical dementia rating sum of boxes; CSF, cerebrospinal fluid; IQR, interquartile range; MCI‐AD, mild cognitive impairment‐Alzheimer's disease; MMSE, Mini‐Mental State Examination; p‐tau181, phosphorylated tau 181; TMEM119, transmembrane protein 119; t‐tau, total tau.

* Median (IQR); *n* (%).

** Wilcoxon rank‐sum exact test, Pearson's chi‐squared test, Wilcoxon rank‐sum test.

### 3.5 ROC analysis of CSF TMEM119

**FIGURE 3:**
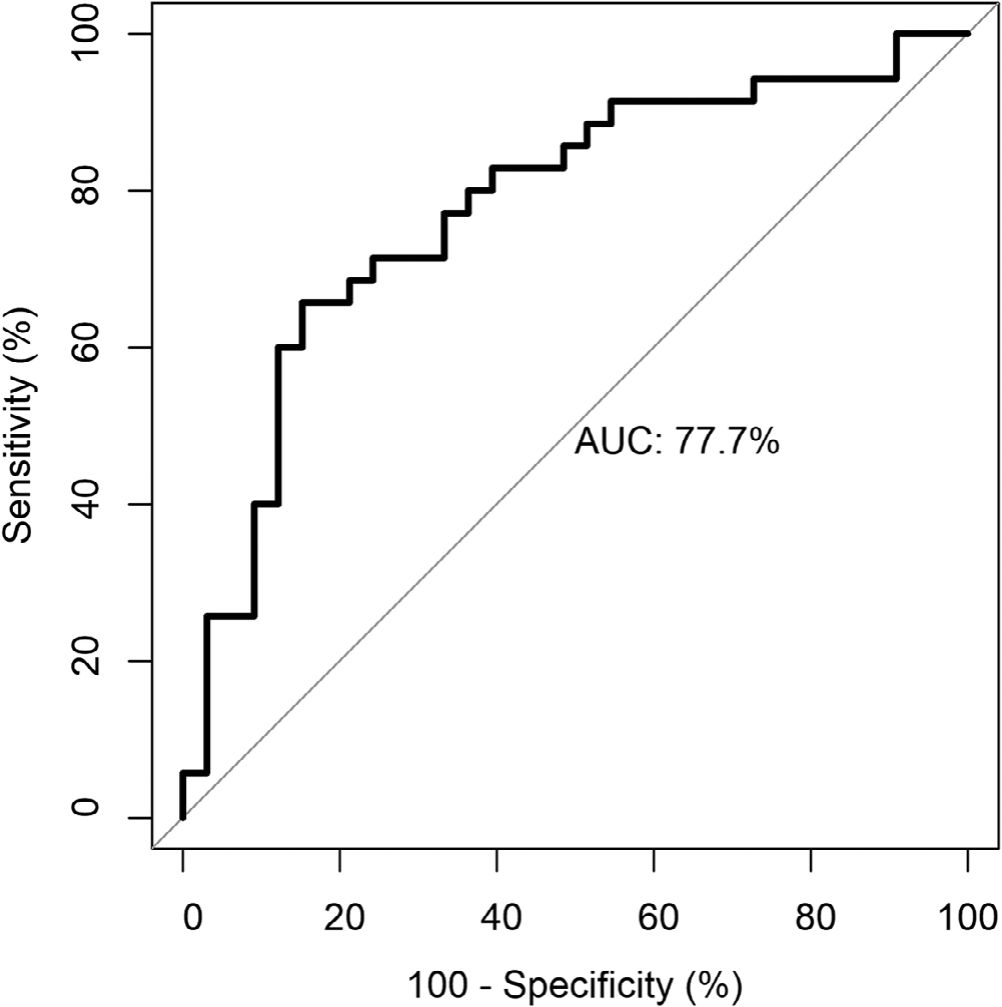
ROC curve for CSF TMEM119 when used to discriminate AD patients from controls. AD, Alzheimer's disease; AUC, area under the curve; CSF, cerebrospinal fluid; ROC, receiver operating characteristic; TMEM119, transmembrane protein 119; t‐tau, total tau.

A ROC analysis was performed in order to determine the potential of CSF TMEM119 to discriminate between the overall AD and control groups (Figure 3). Using Youden's Index, the optimal cut‐off value was determined to be 2.596 ng/mL, corresponding to a specificity of 84.8% and a sensitivity of 65.7%. The results of ROC analysis for the discrimination between the AD and other diagnostic groups may be found in Figure S6 and Table S5. The lowest area under the curve (AUC) was for the discrimination between AD and ALS (0.618), while the AUC for the discrimination between AD and all other diagnostic groups was 0.721.

## 4 DISCUSSION

In this study, we report on the measurement of CSF levels of the microglial marker TMEM119. Testing of assay performance demonstrated results indicative of high reliability and stability. Following validation, the ELISA was used to determine the levels of TMEM119 in the CSF of patients across six diagnostic groups. Elevated levels of CSF TMEM119 were seen in the AD group compared to the control, CM, and FTLD groups. It has been shown that *TMEM119* mRNA expression can be increased in the frontal cortex of AD brains compared to non‐AD brains.^18^ So far, immunohistochemistry staining has shown either no significant difference in protein expression of TMEM119 between AD and non‐AD brains or even a reduction in the number of TMEM119‐positive cells in AD brains.^18^, ^23^, ^24^, ^25^ A recent study using AD mouse models showed that the expression of TMEM119 seems to be spatiotemporally regulated, with reduced expression in microglia in close proximity to Aβ plaques and time‐lapse imaging showing a gradual decrease of TMEM119 fluorescence intensity in the presence of Aβ.^38^ It has been proposed that TMEM119 is part of the transcriptional signature for homeostatic microglia, with a downregulation being observed in DAM states.^21^, ^22^ TMEM119 deficiency has been shown to lead to an accelerated transition of microglia to a DAM state, whereas overexpression led to ameliorated cognitive deficits and a reduction in Aβ deposition in an AD mouse model.^38^

It has been suggested that the discrepancy between mRNA and protein expression determined by immunohistochemistry at the tissue level may be explained by the cleavage of the protein into extracellular and intracellular domain fragments, resulting in the secreted extracellular domain becoming detectable in CSF.^39^ The upregulation of mRNA and subsequent cleavage of the protein into intra‐ and extracellular domains could account for the increase in TMEM119 that was observed in the CSF of AD patients. An alternative explanation could be the degeneration of microglia resulting in an increased release of TMEM119 into the interstitial fluid and, subsequently, the CSF. This interpretation is supported by evidence of microglial degeneration in certain brain regions at later Braak stages of AD.^40^ Changes to levels of CSF TMEM119 may therefore be indicative of a transition away from a homeostatic state. In future experiments, it would be advantageous to determine which exact epitopes of TMEM119 are detected in the CSF in order to gain a better understanding of the measured protein (fragment).

Interestingly, the second‐highest TMEM119 CSF levels were found in the ALS group corroborating data from a recent study showing an increase in TMEM119‐positive cells in the brains of patients with ALS compared to controls.^26^

When the AD group was stratified into ADD and MCI‐AD subgroups, higher levels of CSF TMEM119 were seen in both of the subgroups when compared to the controls. The observation of higher levels in not only the ADD group but also the MCI‐AD group may indicate that changes in microglial activity already occur in earlier stages of the disease. This notion is supported by evidence that proinflammatory proteins associated with microglia have been demonstrated to be elevated in MCI.^41^ Levels of sTREM2, another microglial marker, have also been shown to be increased in MCI‐AD and even in presymptomatic gene carriers, indicating early microglial changes.^15^, ^42^

As CSF levels of TMEM119 were elevated in the AD cohort, we analyzed the association between CSF TMEM119 and the ATN biomarkers CSF t‐tau, p‐tau181, and Aβ1–42.43 CSF levels of TMEM119 were found to be weakly associated with CSF levels of t‐tau and p‐tau181 but not Aβ1–42 in the overall cohort. The positive association with t‐tau and p‐tau181 in the overall AD group support a potential link between neuroinflammation and the processes of neurodegeneration and tau pathology and mirrors what has been observed in several studies on sTREM2.^14^, ^41^ However, the weak relationship also reflects the differences between the neuroinflammatory microglia marker TMEM119 and the established AD biomarkers. It has been shown that tau pathology can induce microglial activation, and it has been additionally suggested that activated microglia may lead to an exacerbation of tau pathology by damaging axons and dendrites.^44^, ^45^ Examining biomarkers for both tau pathology and microglia together may therefore help to give a clearer picture of pathological processes occurring in neurodegenerative diseases. Analysis of the individual MCI‐AD and ADD subgroups revealed a significant relationship between CSF TMEM119 and CDR‐SOB and CSF t‐tau in the ADD subgroup, but not in the MCI‐AD subgroup. The discrepancy between the subgroups and overall AD cohort may be due to the small sample size of the subgroups.

ROC analysis and the optimization of the Youden Index was used to calculate a cut‐off value of 2.596 ng/mL for the discrimination between patients with AD and controls. A sensitivity of 65.7% and specificity of 84.8% were determined when using this concentration. The AUC was determined to be 0.777. ROC analysis revealed a lower AUC for CSF TMEM119 than what was previously reported for core ATN biomarkers,^46^ indicating that on its own CSF TMEM119 may not provide substantial diagnostic value. The AUC was 0.721 for the discrimination between AD and all other diagnostic groups and was the lowest (0.618) for the discrimination between AD and ALS, underlining that CSF TMEM119 may not be suitable for differentiating between different neurodegenerative diseases. However, a recent study evaluating a variety of CSF and plasma biomarkers found that protein aptamers of TREM2 also showed a predictive ability for AD status, supporting the idea that microglial markers may have additional value in the differential diagnosis of AD.^47^ In future analyses, it may be beneficial to evaluate the discrimination potential of CSF TMEM119 when used in conjunction with other established biomarkers to evaluate whether the predictive power could be improved. Furthermore, CSF TMEM119 could be used as an objective readout on microglia activity/stability in AD therapeutic studies and the monitoring of AD patients already receiving US Food and Drug Administration‐ and European Medicines Agency‐approved therapies, complementing the panel of fluid neuronal and astrocytic markers currently in use. Further validation of CSF TMEM119 could be done with comparisons to *post mortem* data, and it will be important in future studies to assess the relationship of TMEM119 with other microglial biomarkers such as sTREM2 and TSPO PET.^15^, ^42^, ^48^

The strengths of our study include (i) the validation and application of an easy‐to‐use ELISA kit to measure CSF levels of the microglial marker TMEM119, (ii) the measurement of CSF TMEM119 levels in patients with a variety of neurodegenerative diseases, and (iii) the comparison of CSF levels of TMEM119 with other AD biomarkers.

Some limitations include having a lower number of MCI patients compared to those with ADD and a lack of longitudinal samples that would allow for the observation of changes in CSF TMEM119 in the same patients over the course of disease. The lack of cognitive assessment and measurement of AD biomarkers for patients in all diagnostic groups represents another limitation. It is also important to note that although TMEM119 is expressed in the CNS predominantly by microglia, it is also known to be expressed in several peripheral tissues, such as bone and brown adipose tissue.^49^, ^50^ While it is assumed that the group differences in CSF are driven by microglial expression, the development of a blood‐based assay sensitive enough to detect TMEM119 would help to exclude the possibility that they were influenced by peripheral expression.

In conclusion, we observed increased CSF TMEM119 levels in AD patients at both early MCI and later ADD stages compared to controls, indicating that it may have use as a fluid biomarker for microglia status in AD. The measurement of TMEM119 and other microglial markers could be used to expand upon the current ATN biomarkers and provide valuable information on inflammation, another pertinent aspect of AD pathology.

## CONFLICT OF INTEREST STATEMENT

Mona Laible received lecture fees from AstraZeneca and was a member of an advisory board of Servier, outside the submitted work. Hayrettin Tumani received honoraria as a consultant/speaker and/or for events sponsored by Alexion, Bayer, Biogen, Bristol Myers Squibb, Celgene, Diamed, Fresenius, Fujirebio, GlaxoSmithKline, Horizon, Janssen‐Cilag, Merck, Novartis, Roche, Sanofi‐Genzyme, Siemens, Teva, and Viatris. No conflicts are relevant to the present study. All other authors report no competing interests.

## CONSENT STATEMENT

The study was approved by the Ethics Committee of Ulm University (approval number 20/10) and conducted following the Declaration of Helsinki. All patients or their legal proxies gave their written informed consent for inclusion in the study.

## Supporting information



Supporting information

Supporting information
