# Supplementary material for: Increased transmembrane protein 119 (TMEM119) levels in the cerebrospinal fluid of patients with mild cognitive impairment due to Alzheimer's disease suggest early microglial involvement
Source: Alzheimers Dement (Amst). 2025 Dec 31;18(1):e70240. doi: 10.1002/dad2.70240 (PMC12756045; doi:10.1002/dad2.70240)
Supplement: Supplementary file 1 — Supporting information [file DAD2-18-e70240-s002.zip › Supplementary Table 5.docx]

| **Supplementary Table 5.** ROC curve analysis for CSF TMEM119 when used to discriminate AD patients from other groups. | | | | |
| --- | --- | --- | --- | --- |
|  | AUC | Threshold (ng/ml) | Specificity | Sensitivity |
| AD vs all groups* | 0.721 | 2.546 | 70.5% | 71.4% |
| AD vs ALS | 0.618 | 2.269 | 48.5% | 80.0% |
| AD vs CM | 0.762 | 2.450 | 79.2% | 72.3% |
| AD vs FTLD | 0.762 | 2.524 | 82.1% | 71.4% |
| AD vs LBD | 0.695 | 2.374 | 66.7% | 77.1% |
| *All groups: control + ALS + CM + FTLD + LBD groups  Abbreviations: AD, Alzheimer’s disease; ALS, amyotrophic lateral sclerosis; CM, cerebral microangiopathy; CSF, cerebrospinal fluid; FTLD, frontotemporal lobar degeneration; IQR, interquartile range; LBD, Lewy body diseases; ROC, receiver operating characteristic; TMEM119, transmembrane protein 119. | | | | |

Supplementary Table 5: Results from ROC curve analysis for CSF TMEM119 when used to discriminate AD patients from various diagnostic groups. AD, Alzheimer’s disease; ALS, amyotrophic lateral sclerosis; CSF, cerebrospinal fluid; CM, cerebral microangiopathy; FTLD, frontotemporal lobar degeneration; LBD, Lewy body diseases; ROC, receiver operating characteristic; TMEM119, transmembrane protein 119.
