# Supplementary material for: Increased transmembrane protein 119 (TMEM119) levels in the cerebrospinal fluid of patients with mild cognitive impairment due to Alzheimer's disease suggest early microglial involvement
Source: Alzheimers Dement (Amst). 2025 Dec 31;18(1):e70240. doi: 10.1002/dad2.70240 (PMC12756045; doi:10.1002/dad2.70240)
Supplement: Supplementary file 1 — Supporting information [file DAD2-18-e70240-s002.zip › Supplementary Figure 1.docx]

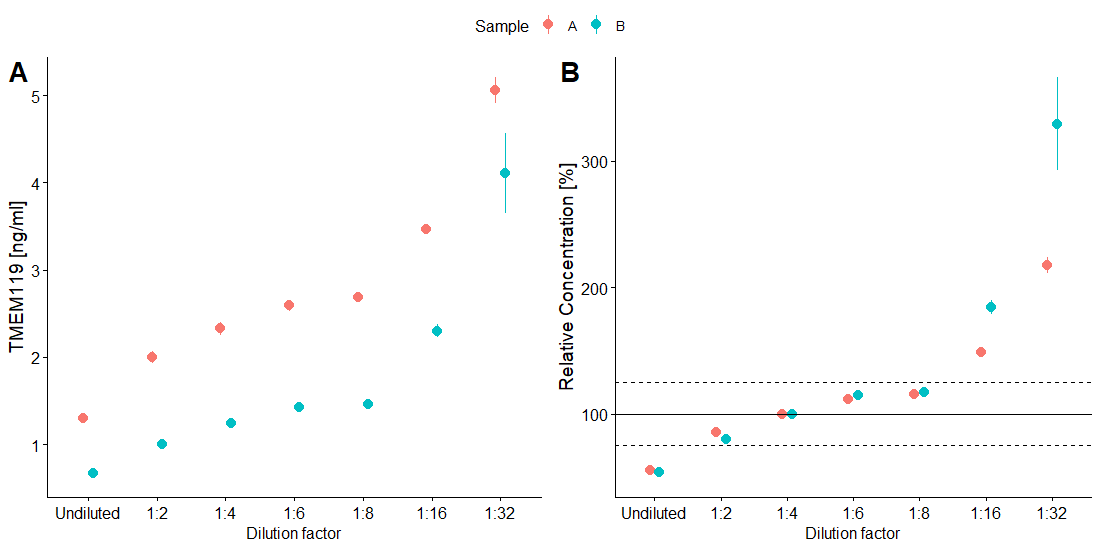


Supplementary Figure 1: Assessment of assay parallelism in two CSF samples. (A) Concentrations of TMEM119 in two CSF samples, back-calculated in order to account for dilution factor. (B) The relative concentration of the different dilutions when compared to the samples diluted 1:4. Dilutions between 1:2 and 1:8 were determined to have an acceptable relative concentration as they fell between 80-120% of the concentration of the samples diluted 1:4. Undiluted samples and those diluted 1:16 and 1:32 fell outside of the acceptable range. CSF, cerebrospinal fluid; TMEM119, transmembrane protein 119.
