# Supplementary material for: Increased transmembrane protein 119 (TMEM119) levels in the cerebrospinal fluid of patients with mild cognitive impairment due to Alzheimer's disease suggest early microglial involvement
Source: Alzheimers Dement (Amst). 2025 Dec 31;18(1):e70240. doi: 10.1002/dad2.70240 (PMC12756045; doi:10.1002/dad2.70240)
Supplement: Supplementary file 1 — Supporting information [file DAD2-18-e70240-s002.zip › Supplementary Figure 2.docx]

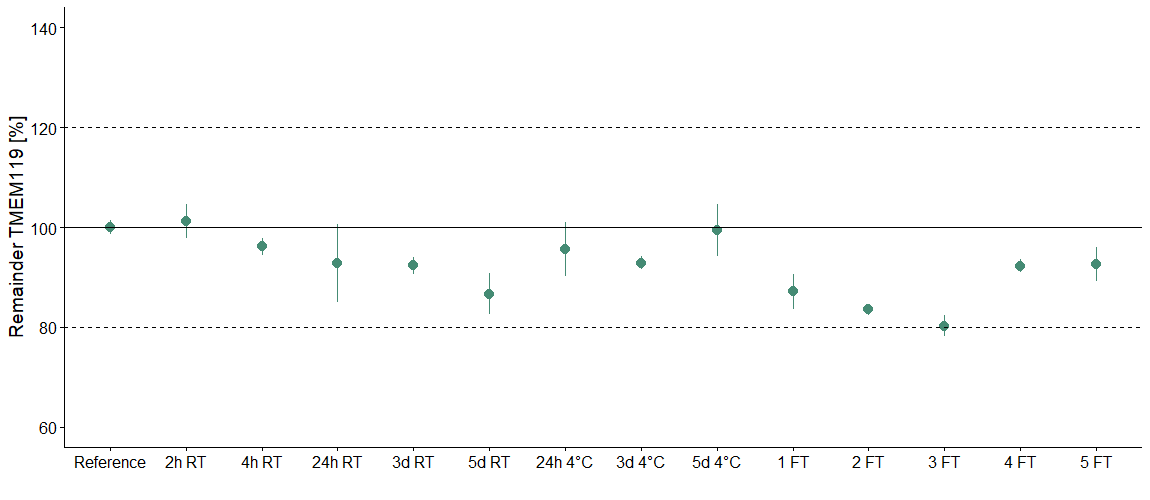


Supplementary Figure 2: Determination of the stability of CSF TMEM119. Equal volumes of CSF were aliquoted and stored at different temperatures prior to being frozen at -80°C. Other aliquots were subjected to additional freeze/thaw cycles when compared to the reference aliquot. Points represent mean CSF TMEM119 levels from samples run in duplicate normalized to the reference sample, with bars representing SD. C, Celsius; CSF, cerebrospinal fluid; d, day; h, hour; FT, freeze-thaw cycle; RT, room temperature; TMEM119, transmembrane protein 119.
