# Supplementary material for: Increased transmembrane protein 119 (TMEM119) levels in the cerebrospinal fluid of patients with mild cognitive impairment due to Alzheimer's disease suggest early microglial involvement
Source: Alzheimers Dement (Amst). 2025 Dec 31;18(1):e70240. doi: 10.1002/dad2.70240 (PMC12756045; doi:10.1002/dad2.70240)
Supplement: Supplementary file 1 — Supporting information [file DAD2-18-e70240-s002.zip › Supplementary Figure 4.docx]

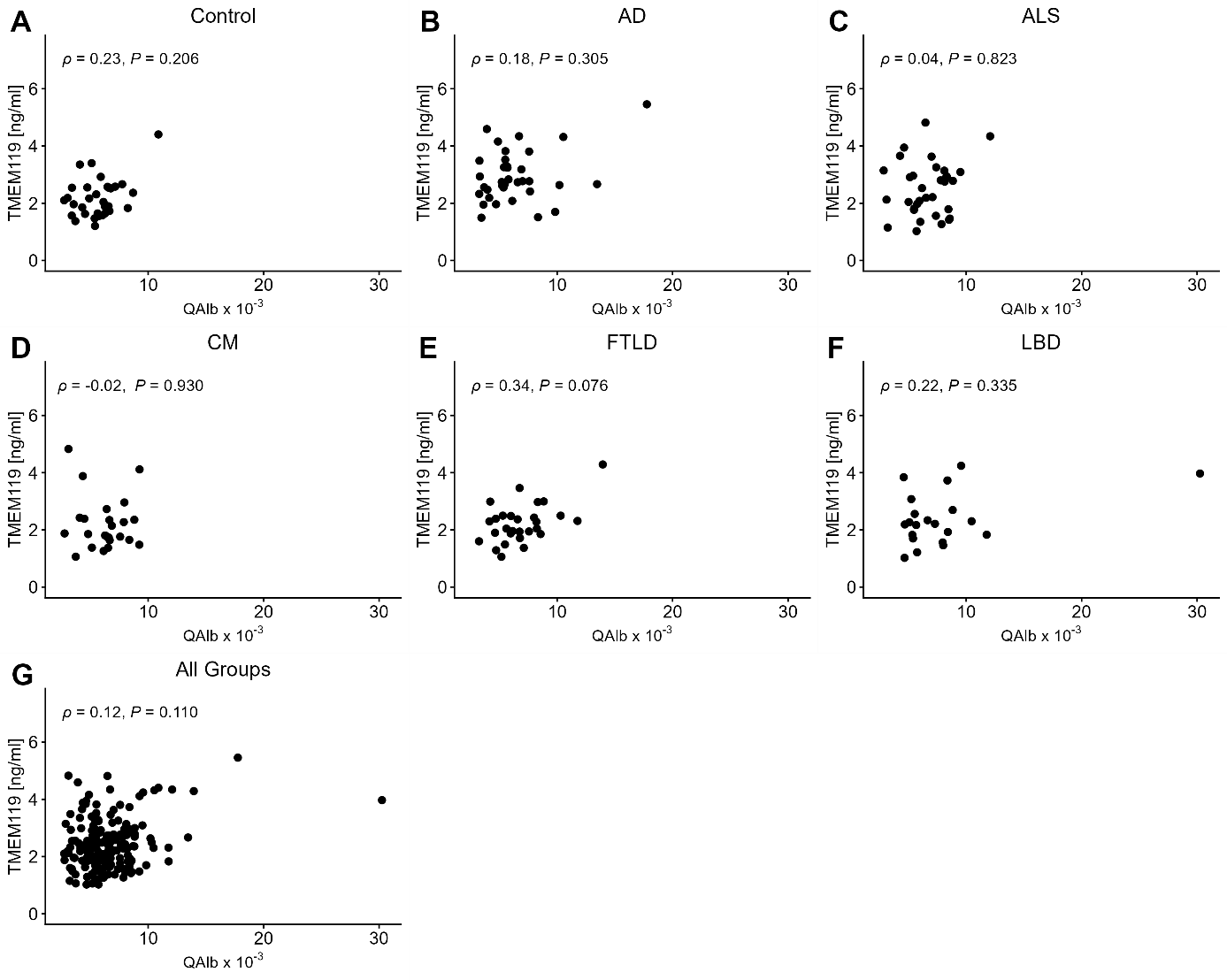


Supplementary Figure 4: Correlation of CSF TMEM119 with QAlb. (A-F) Spearman’s rank correlation showed no significant correlation between TMEM119 and QAlb in any of the individual diagnostic groups (G) Spearman’s rank correlation showed no significant correlation between CSF TMEM119 and QAlb in the overall cohort. AD, Alzheimer’s disease; ALS, amyotrophic lateral sclerosis; CSF, cerebrospinal fluid; CM, cerebral microangiopathy; FTLD, frontotemporal lobar degeneration; LBD, Lewy body diseases; TMEM119, transmembrane protein 119; QAlb, albumin quotient.
