# Supplementary material for: Increased transmembrane protein 119 (TMEM119) levels in the cerebrospinal fluid of patients with mild cognitive impairment due to Alzheimer's disease suggest early microglial involvement
Source: Alzheimers Dement (Amst). 2025 Dec 31;18(1):e70240. doi: 10.1002/dad2.70240 (PMC12756045; doi:10.1002/dad2.70240)
Supplement: Supplementary file 1 — Supporting information [file DAD2-18-e70240-s002.zip › Supplementary Figure 5.docx]

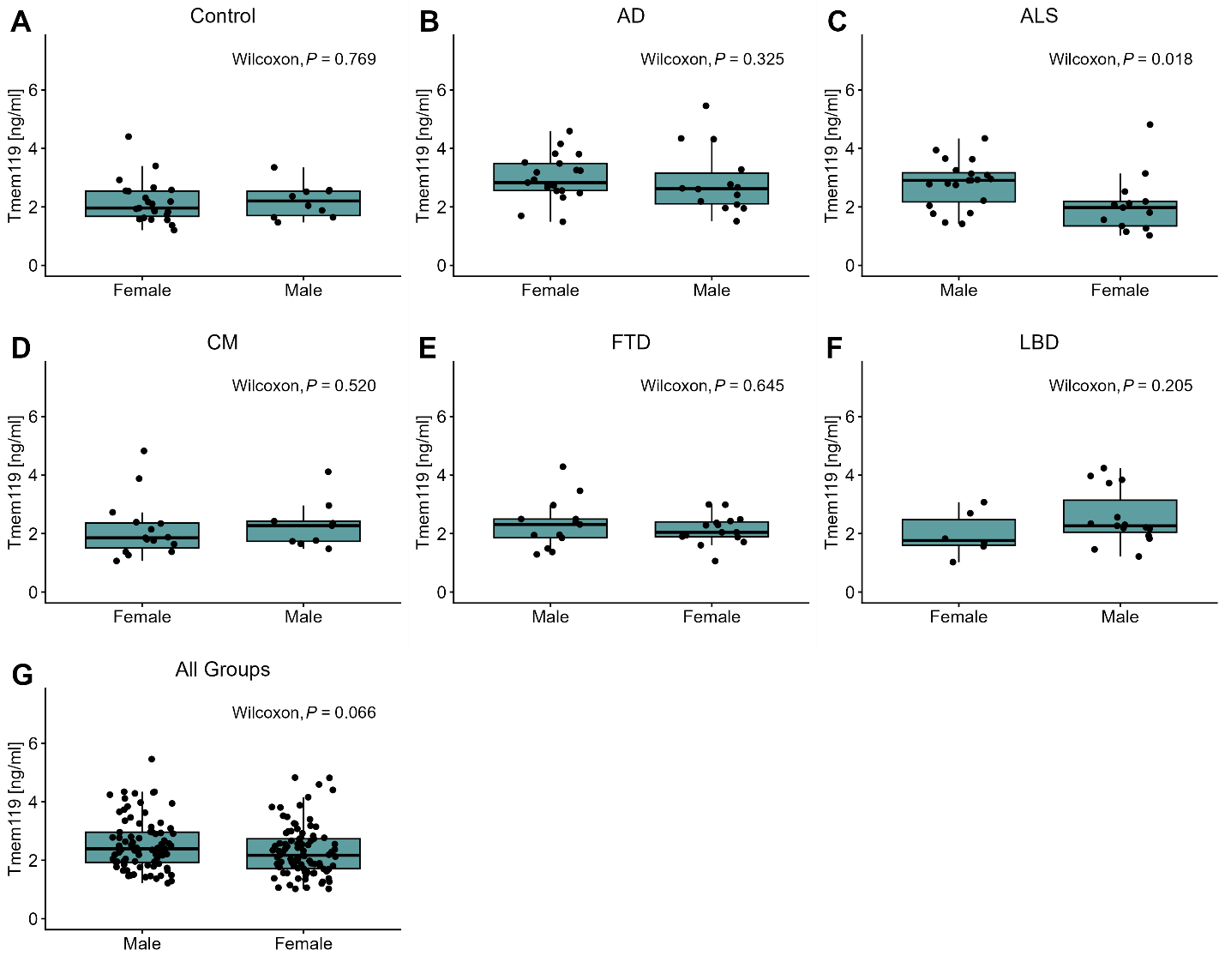


Supplementary Figure 5: Box plot of CSF TMEM119 concentrations stratified by sex and diagnostic group. (A) A Wilcoxon rank sum test revealed no significant difference in TMEM119 levels between males and females in the (A) control (P=0.769), (B) AD (P=0.325), (D) CM (P=0.520), (E) FTLD (P=0.645), and (F) LBD (P=0.205) groups. (C) A significant difference was found between males and females in the ALS group (P=0.018). (G) In the overall cohort, a statistically significant difference was not observed in CSF TMEM119 levels between males and females (P=0.066). Concentrations are displayed as box plots with points representing individual patients. The median and IQR are illustrated, with whiskers extending to ±1.5 × IQR. AD, Alzheimer’s disease; ALS, amyotrophic lateral sclerosis; CSF, cerebrospinal fluid; CM, cerebral microangiopathy; FTLD, frontotemporal lobar degeneration; LBD, Lewy body diseases; TMEM119, transmembrane protein 119.
