# Supplementary material for: Increased transmembrane protein 119 (TMEM119) levels in the cerebrospinal fluid of patients with mild cognitive impairment due to Alzheimer's disease suggest early microglial involvement
Source: Alzheimers Dement (Amst). 2025 Dec 31;18(1):e70240. doi: 10.1002/dad2.70240 (PMC12756045; doi:10.1002/dad2.70240)
Supplement: Supplementary file 1 — Supporting information [file DAD2-18-e70240-s002.zip › Supplementary Figure 6.docx]

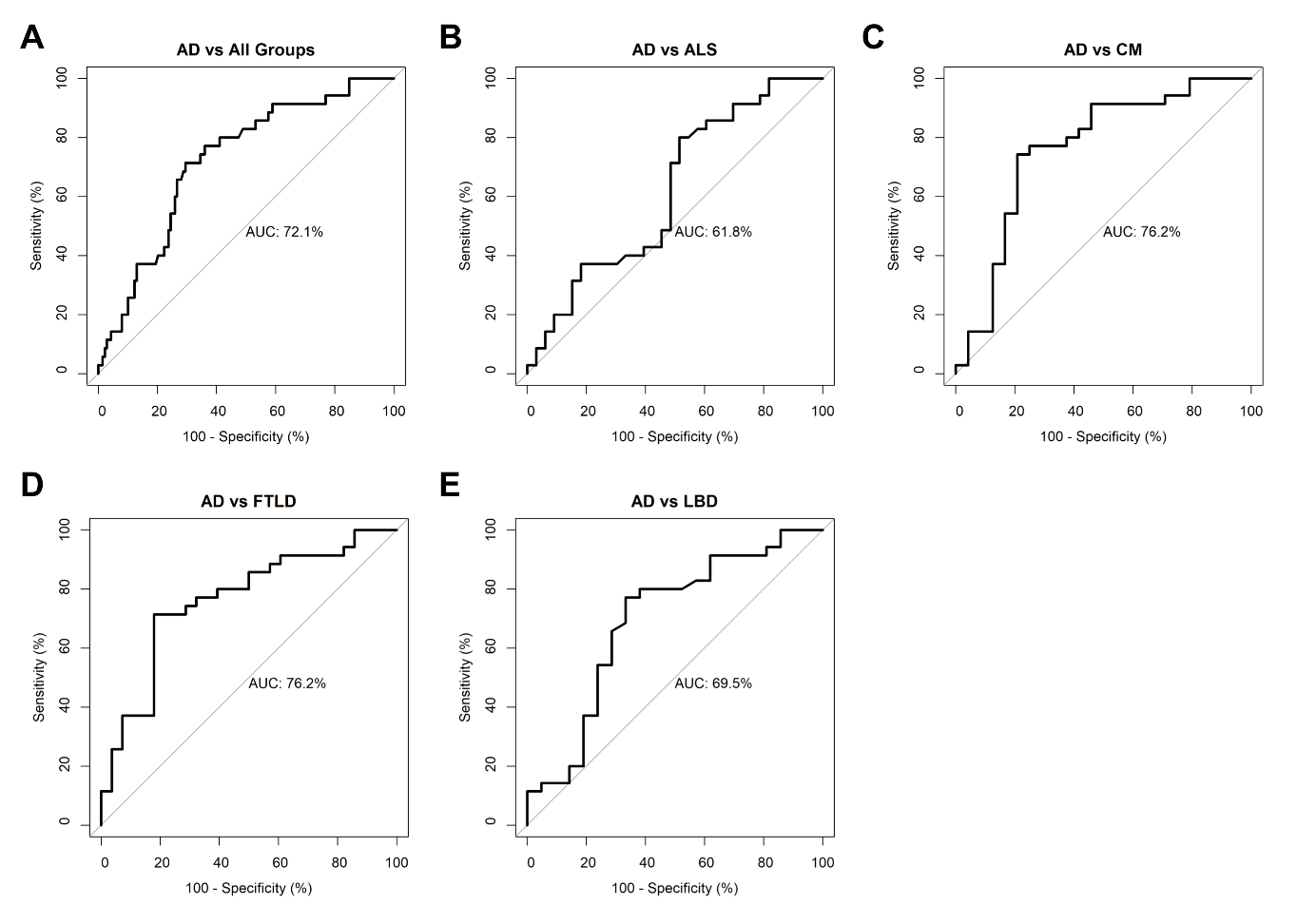


Supplementary Figure 6: ROC curve for CSF TMEM119 when used to discriminate AD patients from (A) all other diagnostic groups including the control group, (B) ALS, (C) CM, (D) FTLD, and (E) LBD. AD, Alzheimer’s disease; ALS, amyotrophic lateral sclerosis; AUC, area under the curve; CM, cerebral microangiopathy; CSF, cerebrospinal fluid; FTLD, frontotemporal lobar degeneration; LBD, Lewy body diseases; ROC, receiver operating characteristic; TMEM119, transmembrane protein 119.
