# Supplementary material for: Increased transmembrane protein 119 (TMEM119) levels in the cerebrospinal fluid of patients with mild cognitive impairment due to Alzheimer's disease suggest early microglial involvement
Source: Alzheimers Dement (Amst). 2025 Dec 31;18(1):e70240. doi: 10.1002/dad2.70240 (PMC12756045; doi:10.1002/dad2.70240)
Supplement: Supplementary file 1 — Supporting information [file DAD2-18-e70240-s002.zip › Supplementary Table 1.docx]

| **Concentration** | **Recovery** |
| --- | --- |
| 300 pg/ml | 91.9% |
| 800 pg/ml | 89.6% |
| 1500 pg/ml | 94.9% |

Supplementary Table 1: Spike and recovery results. CSF samples diluted 1:4 were spiked with three concentrations of the standard TMEM119 protein from the ELISA kit. Recovery was calculated according to the formula described by Andreasson et al^1^. CSF, cerebrospinal fluid; ELISA, enzyme-linked immunosorbent assay; TMEM119, transmembrane protein 119.

*References*:

1. Andreasson U, Perret-Liaudet A, van Waalwijk van Doorn LJC, et al. A practical guide to immunoassay method validation. Front Neurol. 2015;6(Aug):1-8. doi:10.3389/fneur.2015.00179
