# Supplementary material for: Increased transmembrane protein 119 (TMEM119) levels in the cerebrospinal fluid of patients with mild cognitive impairment due to Alzheimer's disease suggest early microglial involvement
Source: Alzheimers Dement (Amst). 2025 Dec 31;18(1):e70240. doi: 10.1002/dad2.70240 (PMC12756045; doi:10.1002/dad2.70240)
Supplement: Supplementary file 1 — Supporting information [file DAD2-18-e70240-s002.zip › Supplementary Table 2.docx]

| Supplementary Table 2. All Groups - ANCOVA (Type III Sum of Squares): | | | | | |
| --- | --- | --- | --- | --- | --- |
| Effect: | Sum of Squares | Df | Mean Square | *F* | *P* value |
| Sex | 0.995 | 1 | 0.995 | 4.223 | 0.041 |
| Group | 4.578 | 5 | 0.916 | 3.887 | 0.002 |
| Residual | 39.334 | 167 | 0.236 |  |  |

Supplementary Table 2: ANCOVA results. An ANCOVA was conducted on log2-transformed CSF TMEM119 levels to control for sex. The results shown in this table are for the ANCOVA performed using data from all six diagnostic groups included in the study. CSF, cerebrospinal fluid; TMEM119, transmembrane protein 119.
