# Supplementary material for: Increased transmembrane protein 119 (TMEM119) levels in the cerebrospinal fluid of patients with mild cognitive impairment due to Alzheimer's disease suggest early microglial involvement
Source: Alzheimers Dement (Amst). 2025 Dec 31;18(1):e70240. doi: 10.1002/dad2.70240 (PMC12756045; doi:10.1002/dad2.70240)
Supplement: Supplementary file 1 — Supporting information [file DAD2-18-e70240-s002.zip › Supplementary Table 3.docx]

| Supplementary Table 3. All Groups - Tukey HSD post hoc test | | | | | | |
| --- | --- | --- | --- | --- | --- | --- |
| Group 1 | Group 2 | Estimate | SE | Df | T ratio | *P* value |
| Control | AD | -0.412 | 0.118 | 167 | -3.489 | 0.008* |
|  | ALS | -0.108 | 0.122 | 167 | -0.891 | 0.948 |
|  | CM | 0.036 | 0.130 | 167 | 0.280 | 1.000 |
|  | FTLD | 0.000 | 0.125 | 167 | 0.000 | 1.000 |
|  | LBD | -0.025 | 0.139 | 167 | -0.177 | 1.000 |
| AD | ALS | 0.303 | 0.119 | 167 | 2.553 | 0.115 |
|  | CM | 0.448 | 0.129 | 167 | 3.484 | 0.008* |
|  | FTLD | 0.412 | 0.123 | 167 | 3.343 | 0.013* |
|  | LBD | 0.387 | 0.136 | 167 | 2.844 | 0.056 |
| ALS | CM | 0.145 | 0.131 | 167 | 1.103 | 0.880 |
|  | FTLD | 0.108 | 0.125 | 167 | 0.866 | 0.954 |
|  | LB | 0.084 | 0.136 | 167 | 0.617 | 0.990 |
| CM | FTLD | -0.036 | 0.135 | 167 | -0.270 | 1.000 |
|  | LBD | -0.061 | 0.147 | 167 | -0.415 | 0.998 |
| FTLD | LBD | -0.025 | 0.141 | 167 | -0.174 | 1.000 |
| Results are averaged over the level of: Sex | | | | | | |

Supplementary Table 3: Post hoc Tukey HSD results. An ANCOVA was conducted on log2-transformed CSF TMEM119 levels to control for sex. The results shown in this table are for the post hoc Tukey HSD test performed following the ANCOVA. AD, Alzheimer’s disease; ALS, amyotrophic lateral sclerosis; CSF, cerebrospinal fluid; CM, cerebral microangiopathy; FTLD, frontotemporal lobar degeneration; LBD, Lewy body diseases; TMEM119, transmembrane protein 119.
