# Supplementary material for: Increased transmembrane protein 119 (TMEM119) levels in the cerebrospinal fluid of patients with mild cognitive impairment due to Alzheimer's disease suggest early microglial involvement
Source: Alzheimers Dement (Amst). 2025 Dec 31;18(1):e70240. doi: 10.1002/dad2.70240 (PMC12756045; doi:10.1002/dad2.70240)
Supplement: Supplementary file 1 — Supporting information [file DAD2-18-e70240-s002.zip › Supplementary Table 4.docx]

| Supplementary Table 4. Control group and AD subgroups - ANCOVA (Type III Sum of Squares): | | | | | |
| --- | --- | --- | --- | --- | --- |
| Effect: | Sum of Squares | Df | Mean Square | *F* | *P* value |
| Sex | 0.019 | 1 | 0.019 | 0.104 | 0.748 |
| Group | 3.149 | 2 | 1.575 | 8.498 | <0.001 |
| Residual | 11.858 | 64 | 0.185 |  |  |

Supplementary Table 4: ANCOVA results. An ANCOVA was conducted on log2-transformed CSF TMEM119 levels to control for sex. The results shown in this table are for the ANCOVA performed using data from the control group and the ADD and MCI-AD subgroups from the AD cohort. AD, Alzheimer’s disease; ADD, Alzheimer’s disease dementia; CSF, cerebrospinal fluid; MCI – AD, Alzheimer’s disease with mild cognitive impairment; TMEM119, transmembrane protein 119.
